# A Red-Emission Fluorescent Probe for Intracellular Biothiols and Hydrogen Sulfide Imaging in Living Cells

**DOI:** 10.3390/molecules29071572

**Published:** 2024-03-31

**Authors:** Yuanfan Wang, Shengxiang Zhang, Tianle Liu, Junning Chen, Bingrui Yuan, Cuntao Lu, Xiumei Bo, Zhou Xu

**Affiliations:** 1 The First Clinical Medical College, Xuzhou Medical University, Xuzhou 221004, China; 2 School of Pharmacy, Xuzhou Medical University, Xuzhou 221004, Chinaxuzhou@xzhmu.edu.cn (Z.X.); 3 Department of Breast Surgery, Xuzhou Central Hospital, Xuzhou 221004, China; 4 School of Basic Medical Sciences, Xuzhou Medical University, Xuzhou 221004, China

**Keywords:** biothiol, fluorescent probe, imaging, red emission, hydrogen sulfide, cysteine, glutathione, tumor

## Abstract

This research centers on the development and synthesis of a longwave fluorescence probe, labeled as **60T**, designed for the simultaneous detection of hydrogen sulfide, cysteine/homocysteine, and glutathione. The probe showcases a swift response, good linearity range, and heightened sensitivity, boasting that the detection limits of the probe for Cys, Hcy, GSH and H_2_S were 0.140, 0.202, 0.259 and 0.396 μM, respectively. Notably, its efficacy in monitoring thiol status changes in live MCF-7 cells is underscored by a substantial decrease in fluorescence intensity upon exposure to the thiol trapping reagent, N-ethyl maleimide (NEM). With an impressive red emission signal at 630 nm and a substantial Stokes shift of 80 nm, this probe exhibits remarkable sensitivity and selectivity for biothiols and H_2_S, indicating promising applications in the diagnosis and surgical navigation of relevant cancers.

## 1. Introduction

Hydrogen sulfide (H_2_S) and small molecule biothiols, including cysteine (Cys), homocysteine (Hcy), and glutathione (GSH), have been identified as pivotal players in the physiological and pathological processes of the biological system [1,2,3,4,5,6,7]. These thiol-containing molecules actively participate in diverse cellular events, including the maintenance of intracellular redox equilibrium, signal transduction cascades, antioxidant defense mechanisms, cancers, etc. Elevated levels of intracellular biothiols/H_2_S have been closely associated with the onset and progression of various diseases, emphasizing the crucial need for their accurate detection in biomedical research [8,9,10]. In the pursuit of detecting hydrogen sulfide (H_2_S) and biothiols, numerous technologies and methodologies have emerged, reflecting the dynamic landscape of analytical methods. Among these, the utilization of fluorescent probes stands out as a particularly promising avenue, showcasing remarkable potential in both in vitro and in vivo applications. This is attributed to their rapid response, exceptional selectivity, high sensitivity, and the ability to facilitate real-time imaging [11,12,13,14]. The ability to specifically target and distinguish between various sulfur-containing species enhances the reliability and applicability of these probes in diverse experimental settings. Moreover, the high sensitivity exhibited by fluorescent probes enhances their capability to detect trace amounts of H_2_S and biothiols. This heightened sensitivity is crucial, especially when dealing with biological samples where target analytes may be present in low concentrations. The ability to achieve reliable detection at such levels broadens the scope of applications, spanning from fundamental research to clinical diagnostics. However, the presence of diverse compounds and biomolecules in biological samples poses a challenge for achieving highly selective detection of total biothiols/H_2_S.

Recent studies have showcased the successful design and application of fluorescent probes for the detection of biological thiols/H_2_S. These methods offer powerful and efficient means for the real-time monitoring of biothiols/H_2_S, holding great promise for profound impacts in the fields of life sciences and medical diagnostics [15,16,17,18,19]. The current availability of fluorescence probes capable of simultaneously detecting hydrogen sulfide, cysteine/homocysteine, and glutathione remains relatively limited. Most probes designed for thiol detection can only identify specific components, lacking the ability to comprehensively detect total biothiols and hydrogen sulfide. Comprehensive tracking of the levels of biothiols and hydrogen sulfide is crucial for in-depth exploration, diagnosis, and treatment of conditions such as tumors [20,21,22,23]. The predominant issue lies in their relatively short maximal emission wavelengths, potentially causing interference with background emission from biomaterials in cells and tissues. This limitation contributes to reduced sensitivity and compromised temporal–spatial resolution. Additionally, although some red-emission or near-infrared (NIR) probes have been developed for biothiol detection, many exhibit small Stokes shifts, posing a disadvantage due to potential self-absorption. On the other hand, NBD-based probes are indeed known to undergo nucleophilic aromatic substitution (SNAr) reactions with suitable nucleophiles. In these reactions, the nucleophile attacks the electron-deficient NBD ring, resulting in the substitution of a leaving group with the nucleophile. These reactions are widely utilized in various chemical and biological applications, including fluorescence labeling of biomolecules, sensor development, and imaging studies [24,25]. Therefore, there remains a considerable demand for probes featuring both long-wavelength emission and a substantial Stokes shift.

Based on the considerations outlined above, we endeavored to address the challenges in thiol detection by designing and synthesizing a longwave fluorescence probe capable of simultaneous detection of hydrogen sulfide, cysteine/homocysteine, and glutathione. This endeavor aims to provide the field of biomedical research with more accurate and comprehensive tools for thiol detection, with the potential to drive advancements and offer new avenues for early disease diagnosis. In the course of this study, we introduce a novel fluorescence probe, designated as **60T**, inspired by the NBD-Cl moiety. Research findings reveal that the **60T** fluorescence probe boasts exceptional sensitivity (48-fold increase), enabling rapid detection of biothiols and H_2_S within an extremely short timeframe (30 s). Notably, the probe demonstrates an outstanding linear range, with detection limits for various biothiols reaching as low as one hundred nanomolar levels. Further, the probe was proved to be capable of detecting the thiols and H_2_S in live MCF-7 and HepG-2 cells. Unlike other probes, our probe has the ability to comprehensively detect both thiol compounds and hydrogen sulfide rather than being limited to one or a few specific types. In certain specific application scenarios, this comprehensiveness may be more practically significant, especially when simultaneous monitoring of multiple thiol-related substances is required. Moreover, organic small molecule fluorescent probes offer advantages such as ease of synthesis, biocompatibility, and tunable fluorescence properties. These attributes underscore the continued relevance and importance of organic small-molecule fluorescent probes in certain applications.

## 2. Results

### 2.1. Design and Synthesis of Probe **60T**

**Scheme 1:**
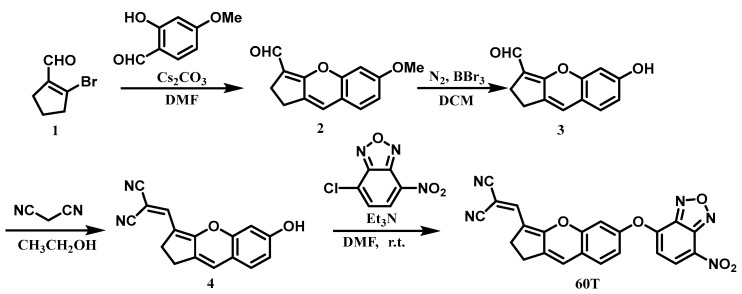
The synthetic route of **60T**.

We designed the probe **60T** constructed by a donor-acceptor type fluorophore and NBD group (Scheme 1). It can be readily synthesized from commercially available and inexpensive starting materials through a four-step reaction sequence, yielding an overall yield of 16%.

*Synthesis and characterization of compound* **2**. At 0 °C, 1.771 g (10 mmol) of compound **1** and 0.913 g (6 mmol) of 2-hydroxy-4-methoxybenzaldehyde were dissolved in 10 mL of DMF. Subsequently, 7.820 g (24 mmol) of cesium carbonate were added, and the reaction mixture was stirred at room temperature for 12 h. The reaction was quenched at 0 °C with dilute hydrochloric acid (2M). The organic layer was extracted with ethyl acetate (3 × 10 mL), and the combined organic layers were dried over anhydrous sodium sulfate. The crude product was then purified by silica gel column chromatography, yielding a yellow solid (1.1 g, 48% yield), known compound. ^1^H NMR (400 MHz, Chloroform-d) δ 10.02 (s, 1H), 7.06 (d, *J* = 8.4 Hz, 1H), 6.72 (d, *J* = 2.4 Hz, 1H), 6.68 (dd, *J* = 8.4, 2.5 Hz, 1H), 6.59 (s, 1H), 3.84 (s, 3H), 2.75–2.69 (m, 4H). ^13^C NMR (101 MHz, Chloroform-d) *δ* 184.5, 164.2, 161.0, 153.0, 137.5, 127.6, 122.4, 116.5, 115.6, 111.4, 101.6, 55.8, 24.4, 23.7 (Figures S1 and S2).
*Synthesis and characterization of compound* **3**. Under nitrogen protection, 457 mg (2 mmol) of compound **2** were dissolved in 4 mL of anhydrous dichloromethane (DCM). At 0 °C, boron tribromide (1.5 mL, 16 mmol) was introduced, and the reaction mixture was stirred at room temperature for 10 h. Quenching the reaction with water at 0 °C was followed by extraction of the organic layer with dichloromethane (3 × 10 mL). The combined organic layers were then dried over anhydrous sodium sulfate. Subsequently, the crude product underwent purification through silica gel column chromatography, yielding a yellow solid (352 mg, 83% yield), known compound. ^1^H NMR (400 MHz, DMSO-*d*_6_) δ 10.17 (s, 1H), 9.90 (s, 1H), 7.18 (d, *J* = 8.7 Hz, 1H), 6.85 (s, 1H), 6.63 (s, 2H), 2.73–2.70 (m, 2H), 2.55–2.53 (m, 2H). ^13^C NMR (101 MHz, DMSO-*d*_6_) *δ* 182.8, 163.4, 159.1, 152.2, 135.6, 128.1, 123.0, 115.2, 113.8, 112.4, 102.7, 23.6, 23.3 (Figures S3 and S4).
*Synthesis and characterization of compound Synthesis of compound* **4**. Compound **3** (352 mg, 1.6 mmol) was dissolved in 3 mL of anhydrous ethanol along with malononitrile (158 mg, 2.4 mmol). The reaction mixture was refluxed at 80 °C with stirring for 3 h. Following rotary evaporation, the residue was extracted with dichloromethane (3 × 10 mL). The combined organic layers were then dried over anhydrous sodium sulfate. The crude product underwent purification via silica gel column chromatography, yielding a red solid (345 mg, 82% yield). ^1^H NMR (400 MHz, DMSO-*d*_6_) δ 10.52 (s, 1H), 7.79 (s, 1H), 7.33 (d, *J* = 8.4 Hz, 1H), 7.22 (s, 1H), 6.78 (d, *J* = 2.2 Hz, 1H), 6.74 (dd, *J* = 8.0, 1.8 Hz, 1H), 2.94–2.92 (m, 2H), 2.86–2.83 (m, 2H). ^13^C NMR (101 MHz, DMSO-*d*_6_) δ 164.9, 160.3, 153.1, 146.4, 134.1, 128.8, 127.7, 117.3, 116.2, 113.9, 113.3, 102.7, 66.0, 54.9, 24.8, 24.5 (Figures S5 and S6). HRMS (ESI^−^) *m*/*z*: [M-H]^−^ Calculated: 261.0770; Found: 261.0661.
*Synthesis and characterization of probe* **60T**. Compound **4** (65 mg, 0.25 mmol) was dissolved in 5 mL of DMF, followed by the addition of 4-chloro-7-nitro-2,1,3-benzoxadiazole (158 mg, 2.4 mmol) and triethylamine (48 μL, 0.35 mmol). The reaction proceeded at room temperature for 4 h. The mixture was then extracted with ethyl acetate (3 × 10 mL), and the combined organic layers were dried over anhydrous sodium sulfate. The crude product underwent purification by silica gel column chromatography, resulting in a dark purple solid (49 mg, 48% yield). ^1^H NMR (400 MHz, DMSO-*d*_6_) δ 8.67 (d, *J* = 8.3 Hz, 1H), 7.84 (s, 1H), 7.67 (d, *J* = 8.5 Hz, 1H), 7.47 (d, *J* = 2.2 Hz, 1H), 7.34 (dd, *J* = 8.5, 2.4 Hz, 1H), 7.30 (s, 1H), 7.00 (d, *J* = 8.3 Hz, 1H), 3.01–2.98 (m, 2H), 2.95–2.92 (m, 2H). ^13^C NMR (101 MHz, DMSO-*d*_6_) δ 163.3, 154.2, 152.4, 151.7, 145.4, 144.4, 138.6, 135.1, 131.1, 129.3, 125.2, 120.6, 117.6, 116.4, 115.3, 114.5, 111.8, 108.6, 69.7, 24.9, 24.7 (Figures S7 and S8). HRMS (ESI^−^) *m*/*z*: Calcd for C_22_H_11_N_5_O_5_ [M-H]^−^: 424.0687; found: 424.0679.

### 2.2. Selective Response of Thiol Probes

**Figure 1:**
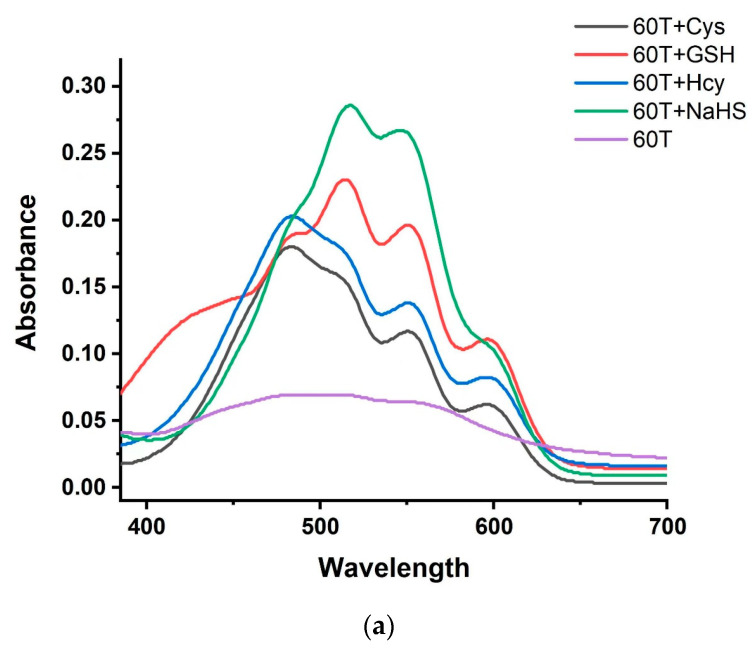

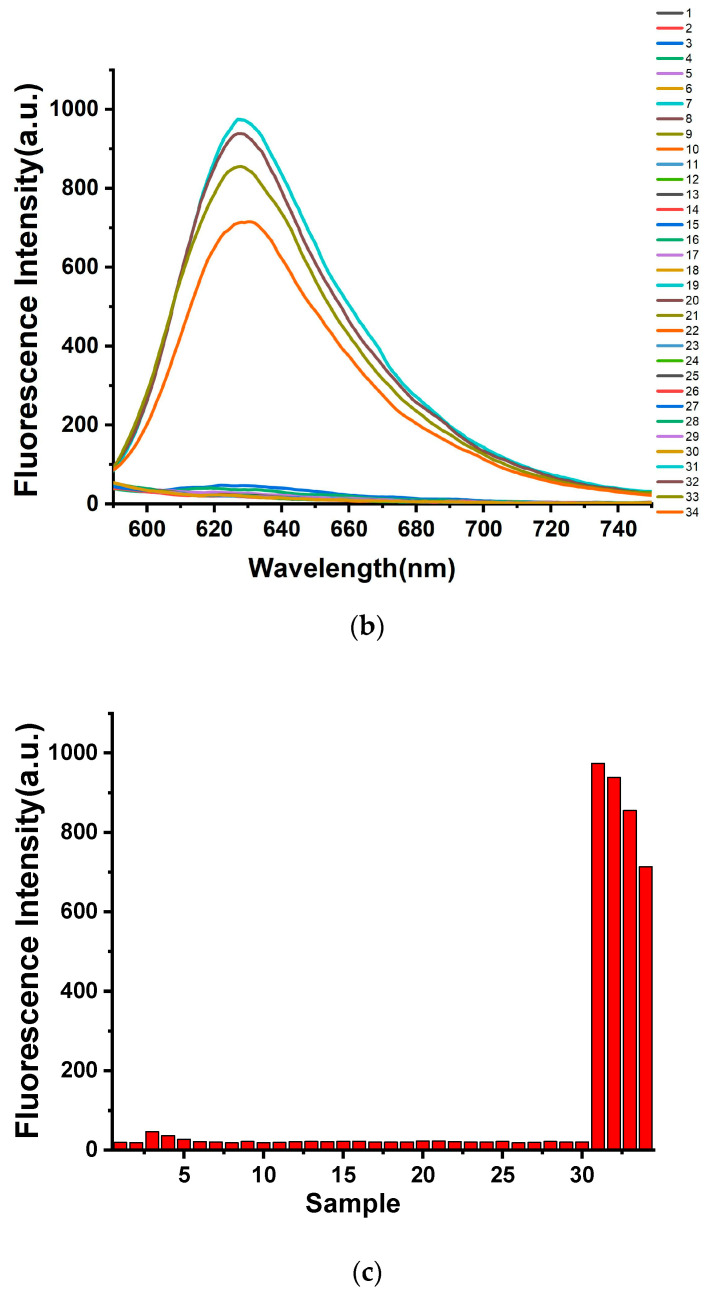
(**a**) Absorption spectra of probe **60T** (10.0 μM) and **60T** + Cys/GSH/Hcy/H_2_S (100 μM) in DMSO–PBS buffer (10 mmol/L, pH = 7.4, 1: 9, *v*/*v*). (**b**) Fluorescence spectra of the probe **60T** (10 μM) in DMSO–PBS buffer (10 mmol/L, pH = 7.4, 1: 9, *v*/*v*) in the presence of various interfering analytes (100 μM). 1. Blank; 2. NaCNS; 3. Na_2_S_2_O_5_; 4. Na_2_S_2_O_4_; 5. Na_2_S_2_O_3_; 6. SO_4_^2−^; 7. Na^+^; 8. K^+^; 9. Ca^2+^; 10. Fe^3+^; 11. F^−^; 12. Cl^−^; 13. Br^−^; 14. HPO_4_^2−^; 15. H_2_PO_4_^−^; 16. NO_3_^−^; 17. HCO_3_^−^; 18. CO_3_^2−^; 19. Mg^2+^; 20. Asp; 21. Pro; 22. Try; 23. Ser; 24. Phe; 25. Trp; 26. Met; 27. His; 28. Lys; 29. Thr; 30. Gly; 31. Cys; 32. Hcy; 33. GSH; 34. NaHS. (**c**) Fluorescence spectra at 630 nm upon the addition of various analytes. E_x_ = 580, E_m_ = 630 nm.

The probe’s distinctive selectivity for a specific analyte among various substances is a crucial characteristic. To explore its unique recognition capabilities, we conducted experiments employing a UV–vis and fluorescence spectrometer. In Figure 1, we present the UV–vis and fluorescence spectral changes observed upon the addition of different analytes (at 10 equiv.) in a DMSO–PBS buffer (10 mmol/L, pH 7.4, 1:9, *v*/*v*). The analytes include NaCNS, Na_2_S_2_O_5_, Na_2_S_2_O_4_, Na_2_S_2_O_3_, SO_4_^2−^, Na^+^, K^+^, Ca^2+^, Fe^3+^, F^−^, Cl^−^, Br^−^, HPO_4_^2−^, H_2_PO_4_^−^, NO_3_^−^, HCO_3_^−^, CO_3_^2−^, Mg^2+^, Asp, Pro, Try, Ser, Phe, Trp, Met, His, Lys, Thr, Gly, Cys, Hcy, GSH, and NaHS. Most of these analytes induced minimal changes or exhibited no changes in the spectra. However, upon the addition of Cys, a substantial enhancement (48-fold) in the emission intensity at 630 nm was observed. Furthermore, subsequent individual additions of Hcy, GSH, and NaHS to the probe **60T** solution led to remarkable enhancements in the emission intensity at 630 nm (47-fold, 43-fold, and 37-fold, respectively). These signal alterations strongly suggest the probe’s potential as a selective chemosensor for thiols and H_2_S.

### 2.3. pH Dependence

**Figure 2:**
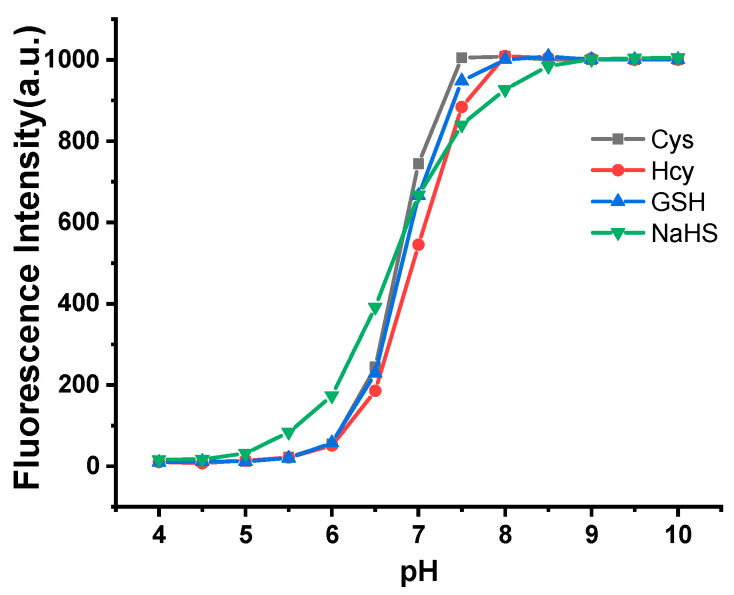
The fluorescence spectra of **60T** (10 μM) in the presence of Cys (100 μM), Hcy (100 μM), GSH (100 μM), and NaHS (100 μM) at different pH ranges.

Due to the fluorescence sensor’s intended application in real cellular samples, it is crucial to assess the impact of pH on detection capabilities, as illustrated in Figure 2. In the presence of biological thiols/H_2_S (100 μM), the fluorescence intensity of probe **60T** exhibits noticeable variations across the pH range of 6 to 10. It is noteworthy that probe **60T** maintains a robust fluorescence intensity for thiols such as Cys under physiological and alkaline conditions. Under neutral and alkaline conditions, the nucleophilicity of thiols and H_2_S is increased. This enhancement in nucleophilicity makes them more reactive towards undergoing addition reactions with the probe **60T**.

### 2.4. Concentration Dependence

**Figure 3:**
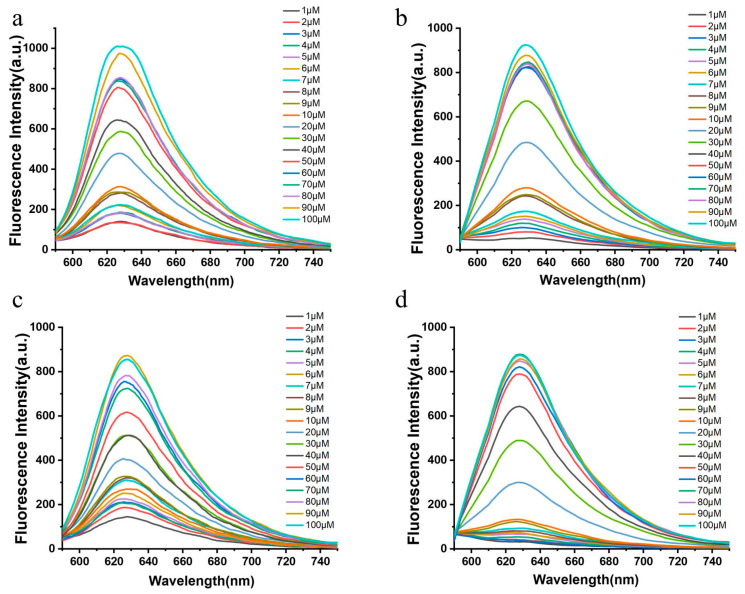
The fluorescence change of **60T** (10 µM) response to various concentrations of Cys (**a**)/Hcy (**b**)/GSH (**c**)/NaHS (**d**) (0–100 µM) after 20 min incubation in a DMSO-PBS buffer (10 mmol/L, pH 7.4, 1:9, *v*/*v*).

The fluorescence spectral responses of probe **60T** (10 μM) to Cys, Hcy, GSH, and NaHS (0~100 μM) were investigated in a DMSO-PBS buffer (10 mmol/L, pH 7.4, 1:9, *v*/*v*). It is evident that the emission peak of probe **60T** at 630 nm gradually intensifies with increasing concentrations of biological thiols (Figure 3).

### 2.5. Detection Limit

**Figure 4:**
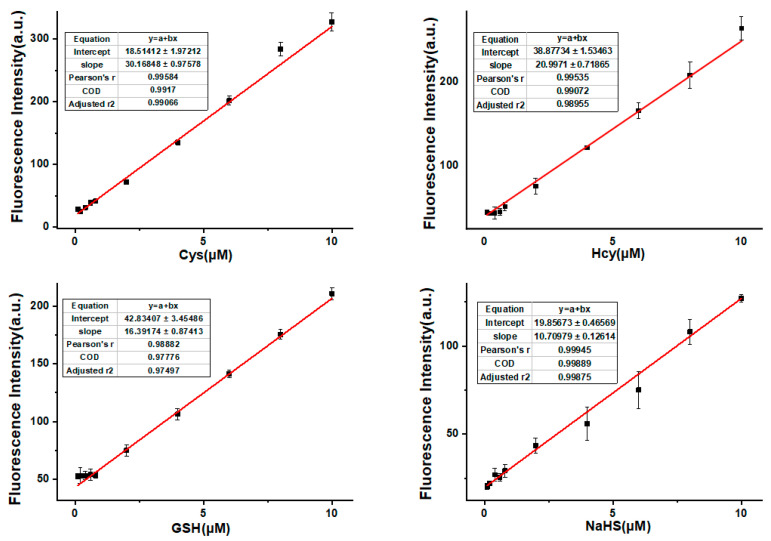
The linearity of the relative fluorescence intensity concerning Cys, Hcy, GSH, and NaHS concentrations was investigated in a DMSO–PBS buffer (10 mmol/L, pH 7.4, 1:9, *v*/*v*) with excitation at 580 nm (slit: 5 nm/5 nm).

To comprehensively evaluate the detection limits of probe **60T** for biological thiols, a systematic analysis was conducted employing a solution of probe **60T** (10 μM). This solution was subjected to a range of concentrations of specific thiols, including Cysteine (Cys), Homocysteine (Hcy), Glutathione (GSH), and Hydrogen Sulfide (NaHS), spanning concentrations from 1 μM to 10 μM. The experimental conditions were meticulously controlled, employing a DMSO–PBS buffer (10 mmol/L, pH 7.4, 1:9, *v*/*v*) to mimic physiological conditions. The fluorescence measurements were carried out with excitation at 580 nm, utilizing slit widths of 5 nm/5 nm, as depicted in Figure 4 for clarity. The obtained data were subsequently subjected to rigorous analysis to reveal the intrinsic relationship between the relative fluorescence intensity at 630 nm and the concentrations of Cys, Hcy, GSH, and NaHS. This analytical approach facilitated the construction of concentration-response curves, elucidating a well-defined linear relationship over a broad concentration range spanning from 0 to 40 mol/L. The calculated detection limits for each thiol, Cys, Hcy, GSH, and NaHS, were determined with meticulous precision. The sensitivity of probe 60T revealed detection limits of 140 nM, 202 nM, 259 nM, and 396 nM for Cys, Hcy, GSH, and NaHS, respectively. This comprehensive detection limit assessment not only establishes the remarkable sensitivity of probe **60T** but also affirms its capability to discern minute concentrations of various biological thiols. These results position probe **60T** as a promising tool for ultrasensitive detection and quantification of biothiols, opening avenues for its application in diverse biological and medical contexts. The methodological rigor employed in this assessment ensures the reliability and accuracy of the determined detection limits, underlining the robustness of probe **60T** in biothiol detection applications.

### 2.6. Time Dependence in Thiol Detection Process

**Figure 5:**
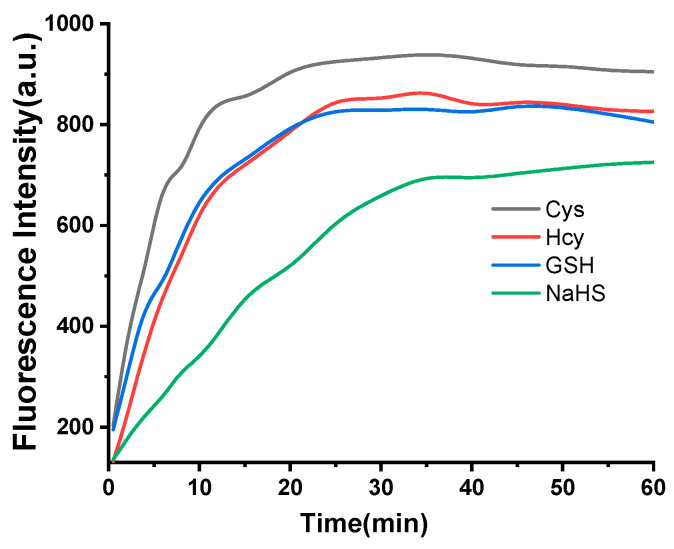
Reaction time profiles of probe **60T** (10 μM) with the addition of 10 equivalents of Cys, Hcy, GSH, and NaHS in DMSO–PBS buffer (1/9, *v*/*v*, pH 7.4). Excitation wavelength (λex) = 580 nm; emission wavelength (λem) = 630 nm.

Over a specified duration, the fluorescence spectra of probe **60T** were systematically monitored in the presence of biological thiols, including Cys, Hcy, GSH, and NaHS, each at a concentration of 100 μM. Notably, Probe **60T** exhibited fluorescence responses to Cys, Hcy, and GSH within approximately 30 s, and the maximum fluorescence intensity for all biological thiols and H_2_S was reached within 30 min (Figure 5).

It is worth mentioning that the response of probe **60T** to Cys reached its maximum fluorescence intensity within 15 min, and the fluorescence intensity remained stable within 30 min. For convenience, all detection times were set to 30 min in the conducted measurements.

### 2.7. Response Mechanism

**Figure 6:**
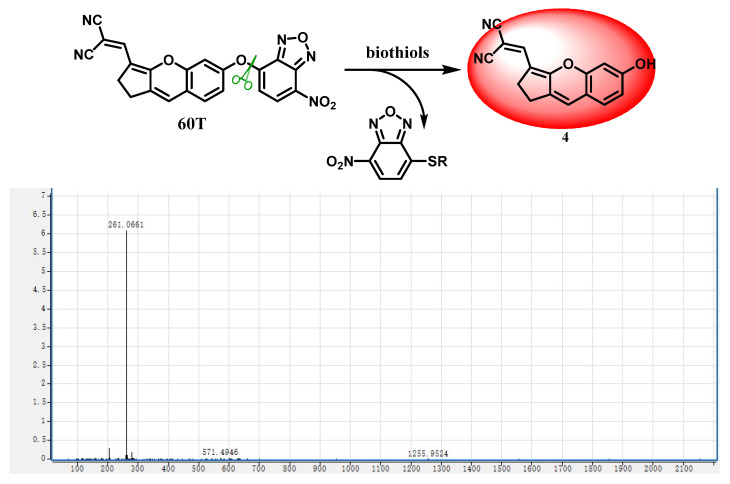
The proposed mechanism of probe **60T** with biothiols and the mass spectra of the mixed solution of **60T** and Cys.

It has been reported that a probe with NBD as a recognition site demonstrated nucleophilic aromatic substitution reaction with biothiols. As shown in Figure 6, biothiols can eliminate the NBD group of **60T**, generating red-emitting fluorophore 4. The mechanism had been preliminarily examined by mass spectra in the mixed solution of 60T and Cys, which proved the production of 4. [M-H]^−^ calculated: 261.0670; found: 261.0661.

### 2.8. Cell Culture and Cytotoxicity Test

**Figure 7:**
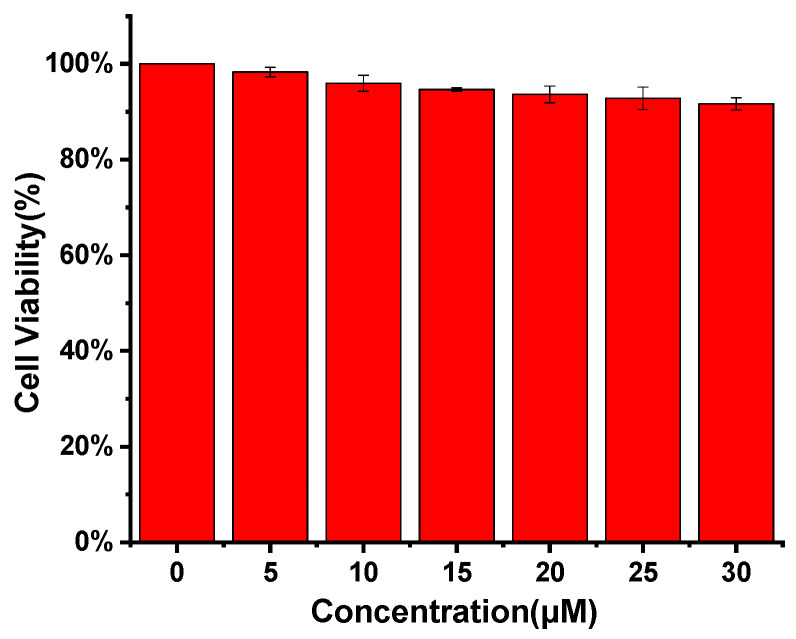
Cell culture and cytotoxicity test.

Before embarking on cell imaging experiments, it is imperative to assess the probe’s toxicity to ensure the reliability of experimental outcomes and the well-being of biological specimens. In the cell viability assay for probe **60T**, the CCK-8 method was employed to evaluate its inhibitory effect on tumor cell growth. A control group was established, and the number of viable cells was determined based on the measured absorbance values. MCF-7 cells were seeded in a 96-well cell culture plate in DMEM medium containing 10% FBS and cultured at 37 °C in a 5% CO_2_ environment. After cellular adhesion, solutions of Probe **60T** at varying concentrations (0, 5, 10, 15, 20, 25, and 30 μM) were added to the cell culture medium, and the cells were incubated for 24 h. Subsequently, CCK-8 solution was added to each well, and the plate was incubated in a cell culture incubator for 1 h. Following color development, absorbance values were measured at 450 nm using a microplate reader. The results indicated that the cell viability remained over 90% within the concentration range of 0–30 μM for the probe (Figure 7). This minimal toxicity demonstrated the probe’s suitability for subsequent cell imaging studies, assuring minimal impact on cell health.

### 2.9. Fluorescence Imaging in Living Cells

**Figure 8:**
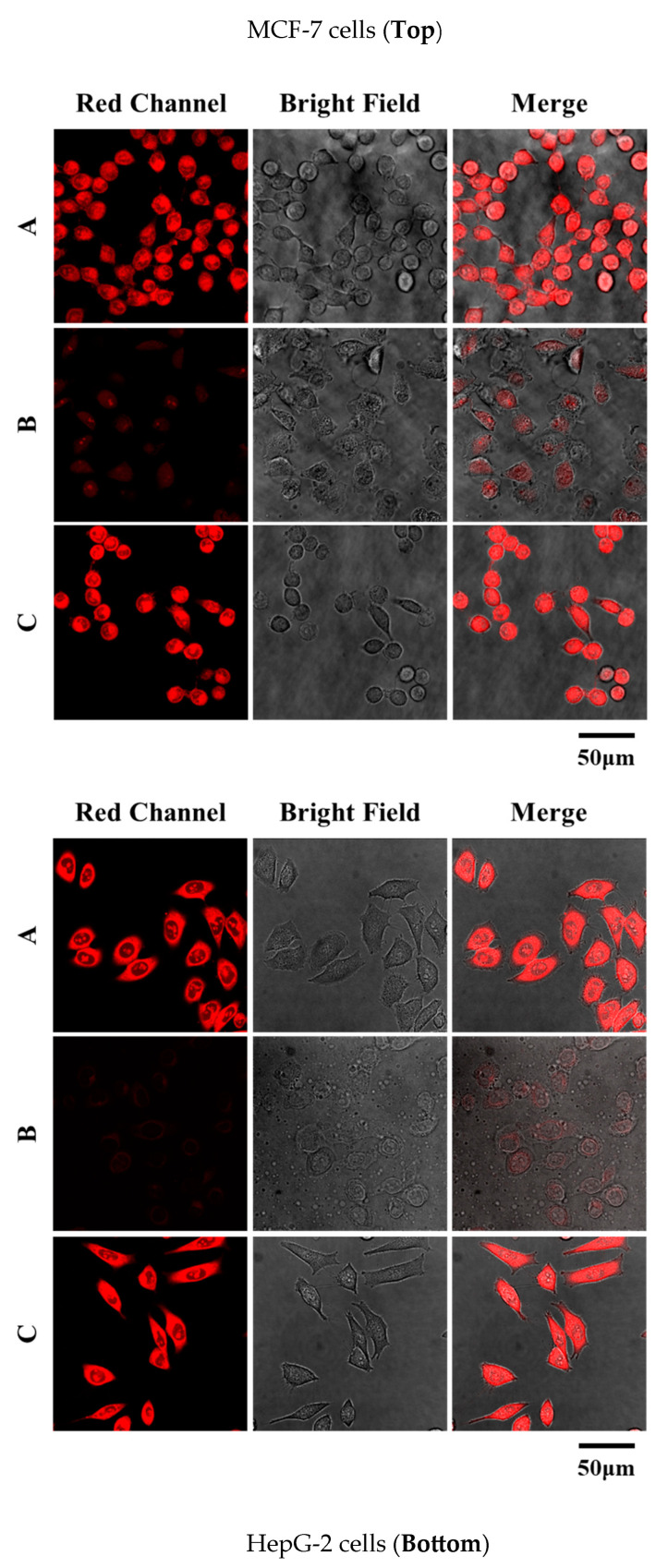
Fluorescence images of MCF-7 cells (**Top**) and HepG-2 cells (**Bottom**). (**A**) Probe **60T** (10 μM) imaged with cells for 30 min; (**B**) cells were treated with N-ethylmaleimide (NEM, a thiol scavenger) (1 mM) for 60 min, followed by incubation with **60T** (10 μM) for 30 min before imaging. (**C**) Cells were treated with NEM (1 mM) for 60 min, then incubated with Cys (100 μM) (representing biological thiols) for 30 min, and finally incubated with **60T** (10 μM) for 30 min.

MCF-7 cells were divided into three groups for imaging. The first group served as the control group and was imaged after incubation with **60T** (10 μM) for 30 min. The second group was the blank control group. Cells were treated with N-ethylmaleimide (NEM, a thiol scavenger) (1 mM) for 60 min, followed by incubation with **60T** (10 μM) for 30 min before imaging. The third group was the experimental group, first treated with NEM (1 mM) for 60 min, then incubated with Cys (100 μM) (representing biological thiols) for 30 min, and finally incubated with **60T** (10 μM) for 30 min. After washing three times with PBS, imaging was performed under a confocal laser scanning microscope with 1ml PBS added. The experimental results revealed clear red fluorescence in the first group, no fluorescence in the second group, and stronger red fluorescence in the third group compared to the first group. The probe also yielded similar results in detecting HepG-2 cells, demonstrating excellent cellular imaging capabilities (Figure 8). The above results indicate that probe **60T** exhibits good cell membrane permeability, enabling the detection of intracellular biological thiols. It can also detect exogenously added biological thiols, making it suitable for labeling thiols in live cells.

## 3. Discussion

In its non-excited state, probe **60T** exhibits non-fluorescence; however, upon reaction with thiols or H_2_S, the NBD moiety dissociates from the molecular structure, releasing the fluorescent core and eliciting a significant fluorescence response. The rapid response, expansive linearity range, and heightened sensitivity of probe 60T set it apart as a promising tool for biothiols and H_2_S detection. The low detection limits for Cys, Hcy, GSH, and H_2_S (0.140, 0.202, 0.259, and 0.396 μM, respectively) underscore its capacity to discern minute concentrations of these analytes. This feature holds significant implications for the probe’s utility in the detection of subtle changes in thiol concentrations within biological systems. The impressive red emission signal at 630 nm, coupled with a substantial Stokes shift of 80 nm, showcases the probe’s capacity for robust signal detection and discrimination. This attribute is crucial for minimizing background interference and enhancing the precision of biothiol and H_2_S detection. The sensitivity and selectivity exhibited by probe **60T** for biothiols and H_2_S highlight its potential in cancer-related applications. The ability to precisely detect these biomolecules suggests promising prospects for the diagnosis and surgical navigation of relevant cancers. The probe’s specificity for biothiols, particularly in the complex cellular milieu, positions it as a valuable tool for advancing our understanding of thiol-related processes in cancer biology. Finally, the observed efficacy of probe **60T** in monitoring thiol status changes in live MCF-7 cells, particularly the substantial decrease in fluorescence intensity upon exposure to the thiol trapping reagent, N-ethylmaleimide (NEM), reinforces its applicability for dynamic thiol studies in living cells. This real-time monitoring capability positions the probe as a valuable asset for investigating thiol-related cellular processes and alterations.

## 4. Materials and Methods

### 4.1. Materials and Instruments

The amino acid stock solution and interference ion stock solution (10.0 mM) were prepared using PBS (pH 7.2–7.4, 1 mM). The amino acids used in the experiment included Asp, Pro, Try, Ser, Phe, Trp, Met, His, Lys, Thr, Gly, Cys, Hcy, and GSH. The probe stock solution (1.0 mM) was prepared in DMSO. In Eppendorf tubes, 20 μL of the probe stock solution were added, followed by the addition of PBS buffer to a total volume of 2 mL. The mixture was incubated at 37 °C in a water bath for 20 min, and fluorescence spectra were recorded. In all tests, both the excitation and emission slit widths for fluorescence spectra were set at 5.0 nm. Reactions were carried out at a constant temperature of 37 °C in a water bath.

The following reagents were used in the experiments: 2-hydroxy-4-methoxybenzaldehyde, cesium carbonate (Cs_2_CO_3_), boron tribromide (BBr_3_), acrylonitrile, 4-Chloro-7-nitro-1,2,3-benzoxadiazole, triethylamine, sodium hydrosulfide (NaHS), and N-ethylmaleimide (NEM).

Nuclear magnetic resonance (NMR) spectra were recorded on a JEOL ECZ400 NMR spectrometer (Akishima, Japan). High-resolution mass spectrometry (HRMS) data were collected using a Bruker Autoflex MALDI-TOF mass spectrometer (Billerica, MA, USA). Absorption spectra were recorded on an Agilent CARY 60 UV-Vis spectrophotometer (Santa Clara, CA, USA). Fluorescence spectra were measured using a Hitachi F-7000 fluorescence spectrophotometer (Tokyo, Japan). Confocal microscopy images of cell fluorescence were obtained using Leica Application Suite X (LAS X, Deerfield, IL, USA). pH values were determined using a PHS-3C digital pH meter (Puchun, Shanghai, China).

### 4.2. Methods

Probe and Stock Solutions Preparation: Dissolve **60T** in dimethyl sulfoxide (DMSO) to prepare a **60T** stock solution (1.0 mM). Dissolve NaHS in phosphate-buffered saline (PBS) buffer (pH 7.4) to obtain an H_2_S stock solution (10.0 mM). Spectral measurements were conducted in the solution with an excitation wavelength set at 580.0 nm, slit widths at 5.5, and a voltage of 650 V. DMSO content: 10%.

Procedure for probe selectivity test: The UV–vis and fluorescence spectral changes are illustrated upon the addition of different analytes (10 equiv.) in a DMSO–PBS buffer (10 mmol/L, pH 7.4, 1:9, *v*/*v*). The analytes include NaCNS, Na_2_S_2_O_5_, Na_2_S_2_O_4_, Na_2_S_2_O_3_, SO_4_^2−^, Na^+^, K^+^, Ca^2+^, Fe^3+^, F^−^, Cl^−^, Br^−^, HPO_4_^2−^, H_2_PO_4_^−^, NO_3_^−^, HCO_3_^−^, CO_3_^2−^, Mg^2+^, Asp, Pro, Try, Ser, Phe, Trp, Met, His, Lys, Thr, Gly, Cys, Hcy, GSH, and NaHS.

Procedure for linear correlation test: The linearity of the relative fluorescence intensity concerning Cys (a), Hcy (b), GSH (c), and NaHS (d) concentrations was investigated in a DMSO–PBS buffer (10 mmol/L, pH 7.4, 1:9, *v*/*v*) with excitation at 580 nm (slit: 5 nm/5 nm).

Cell Culture and Cytotoxicity Test: MCF-7 cells were seeded in a 96-well cell culture plate in DMEM medium containing 10% FBS and cultured at 37 °C in a 5% CO_2_ environment. After cellular adhesion, solutions of probe 60T at varying concentrations (0, 5, 10, 15, 20, 25, and 30 μM) were added to the cell culture medium, and the cells were incubated for 24 h. Subsequently, CCK-8 solution was added to each well, and the plate was incubated in a cell culture incubator for 1 h. Following color development, absorbance values were measured at 450 nm using a microplate reader.

Procedure for confocal imaging: MCF-7 and HepG-2 cells were obtained from the Chinese Academy of Sciences Cell Bank and cultured in high-glucose DMEM medium supplemented with 10% fetal bovine serum at 37 °C in a 5% CO_2_ environment. Cell viability of **60T** (0–30 μM) was assessed using the CCK-8 assay.

Both MCF-7 cells and HepG-2 cells were divided into six groups: the first group served as the control, imaged after incubation with **60T** (10 μM) for 30 min. The second group was the blank control. The following four experimental groups were pre-incubated with NEM (1 mM) for 60 min, followed by separate incubations with Cys (100 μM), Hcy (100 μM), GSH (100 μM), and NaHS (100 μM) for 30 min each. Finally, cells were treated with **60T** (10 μM) for 30 min. After washing three times with PBS, imaging was performed using a confocal laser scanning microscope.

## 5. Conclusions

In summary, we successfully designed and synthesized a fluorescent probe dedicated to the detection of biothiols and H_2_S. The probe showcases notable sensitivity and selectivity for biothiols and H_2_S, presenting a red emission signal at 630 nm. pH dependence experiments have verified the probe’s effectiveness under biological pH conditions. Importantly, we applied the probe to visualize endogenous biothiols in living MCF-7 cells and HepG-2 cells, revealing excellent fluorescent imaging performance. This underscores promising applications in the diagnosis and surgical navigation of relevant cancers.

## Data Availability

Data are contained within the article and Supplementary Materials.

## Supplementary Materials

The following supporting information can be downloaded at https://www.mdpi.com/article/10.3390/molecules29071572/s1, Figures S1–S8: NMR spectra copies compounds **2**–**4** and **60T**.
