# Supplementary material for: A Red-Emission Fluorescent Probe for Intracellular Biothiols and Hydrogen Sulfide Imaging in Living Cells
Source: Molecules. 2024 Mar 31;29(7):1572. doi: 10.3390/molecules29071572 (PMC11013660; doi:10.3390/molecules29071572)
Supplement: Supplementary file 1 [file molecules-29-01572-s001.zip › molecules-2876523-supplementary.pdf]

Supporting information

A Red-Emission Fluorescent Probe for Intracellular Biothiols and Hydrogen Sulfide Imaging in Living Cells

**Yuanfan Wang [1,†], Shengxiang Zhang [2,†], Tianle Liu [1,†], Junning Chen [2], Bingrui Yuan [1], Cuntao Lu [3,*],
Xiumei Bo [4,*] and Zhou Xu [2]**

[1] The First Clinical Medical College, Xuzhou Medical University, Xuzhou 221004, China; 18021195350@163.com (Y.W.); ltl19816266959@163.com (T.L.); 19916640522@163.com (B.Y.)

[2] School of Pharmacy, Xuzhou Medical University, Xuzhou 221004, China; zsx1667@163.com (S.Z.);
chenjunning2024@163.com (J.C.); xuzhou@xzhmu.edu.cn (Z.X.)

[3] Department of Breast Surgery, Xuzhou Central Hospital, Xuzhou 221004, China

[4] School of Basic Medical Sciences, Xuzhou Medical University, Xuzhou 221004, China

* Correspondence: lct71927@163.com (C.L.); xiumeibo@xzhmu.edu.cn (X.B.)

† These authors contributed equally to this work.

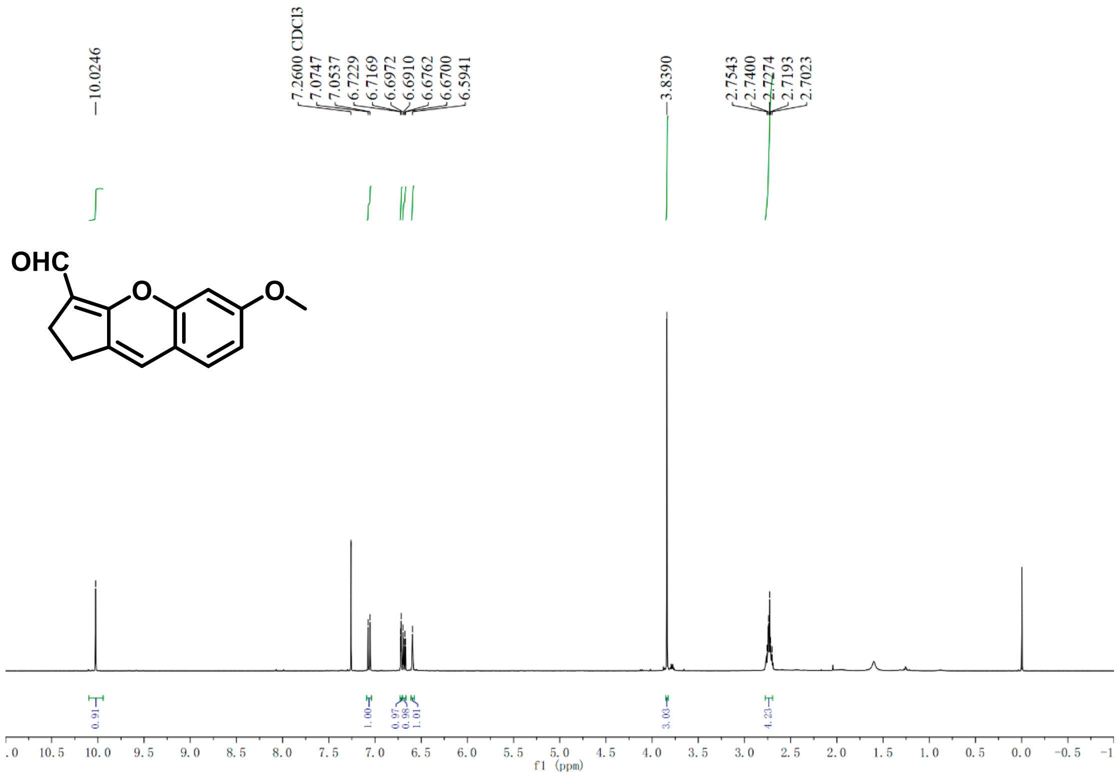

Figure S1. ^{1}H NMR spectrum (400 MHz, Chloroform-d) of **2**

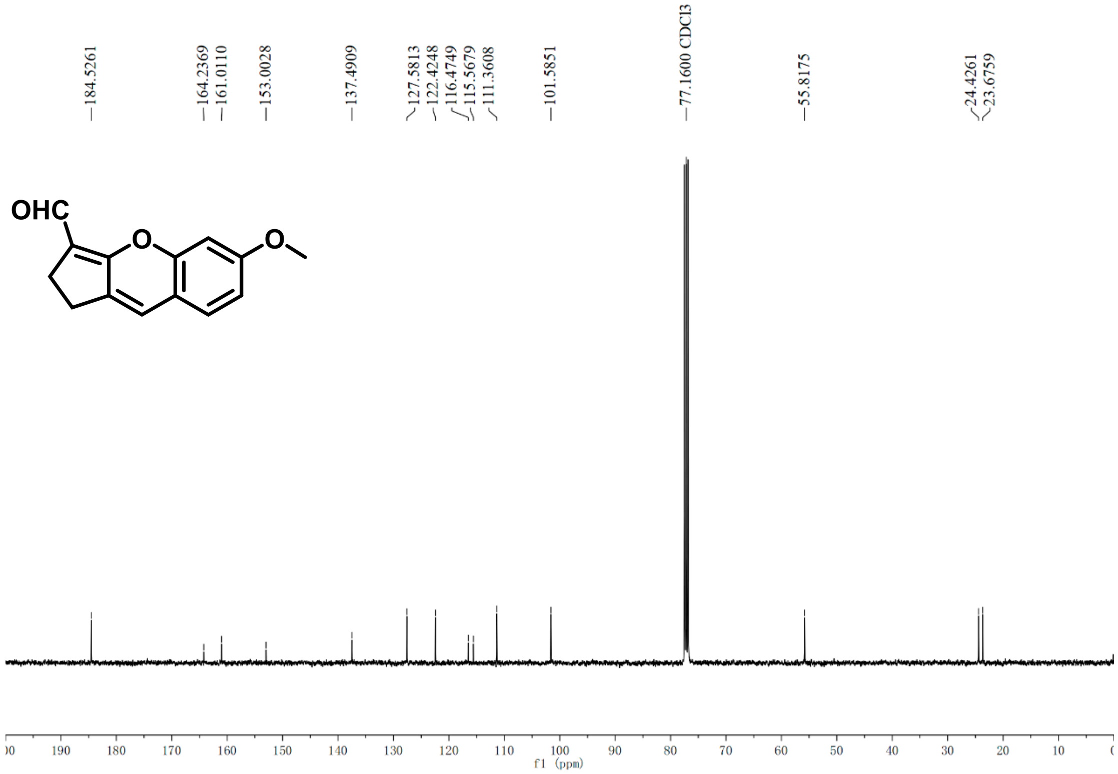

Figure S2. ^{13}C NMR spectrum (101 MHz, Chloroform-d) of **2**.

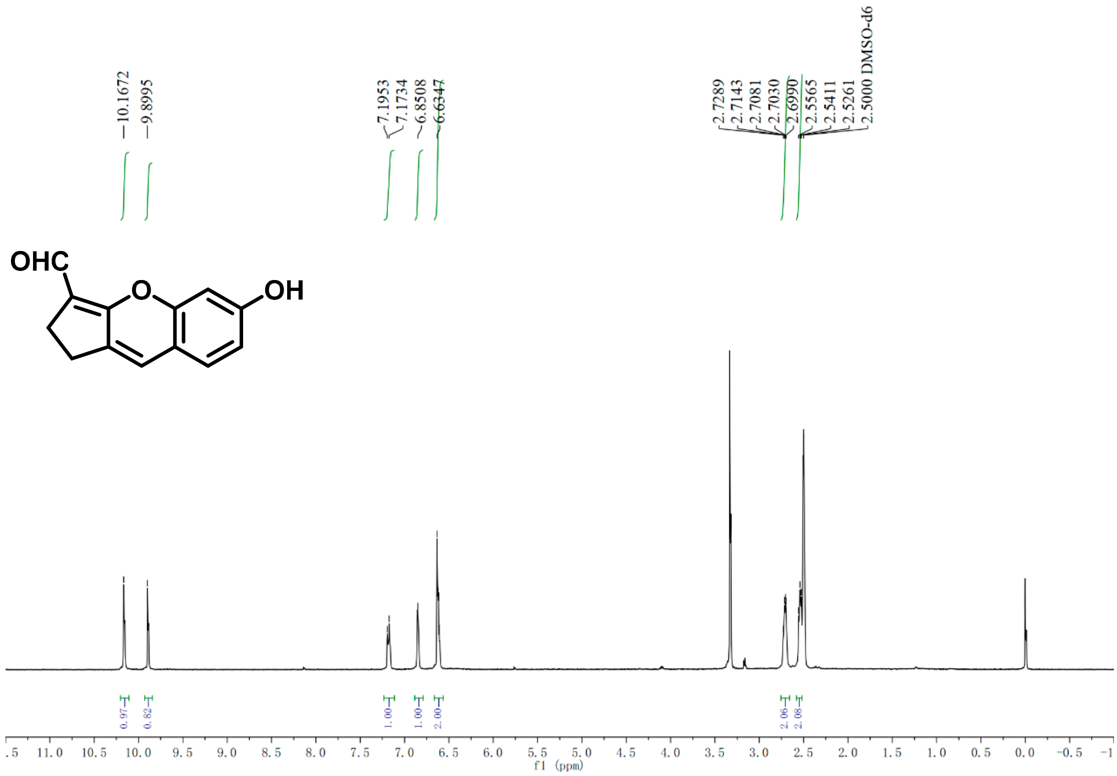

Figure S3. ^{1}H NMR spectrum (400 MHz, DMSO-d6) of **3**

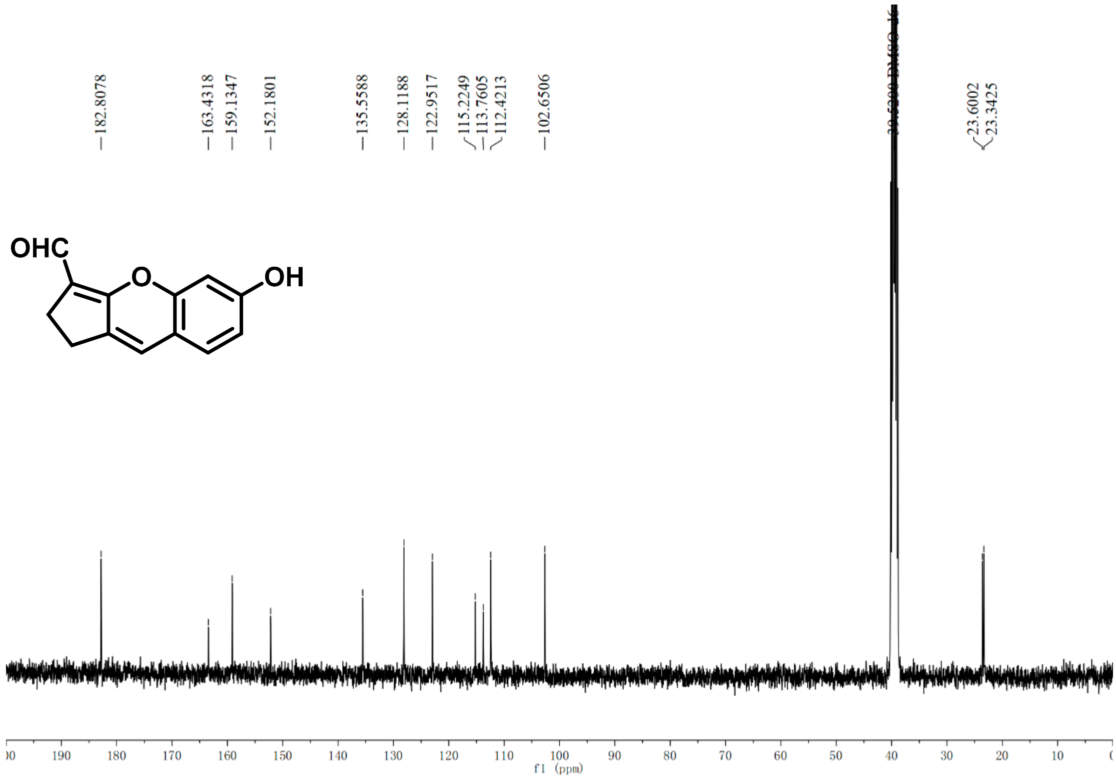

Figure S4. ^{13}C NMR spectrum (101 MHz, DMSO-d6) of **3**.

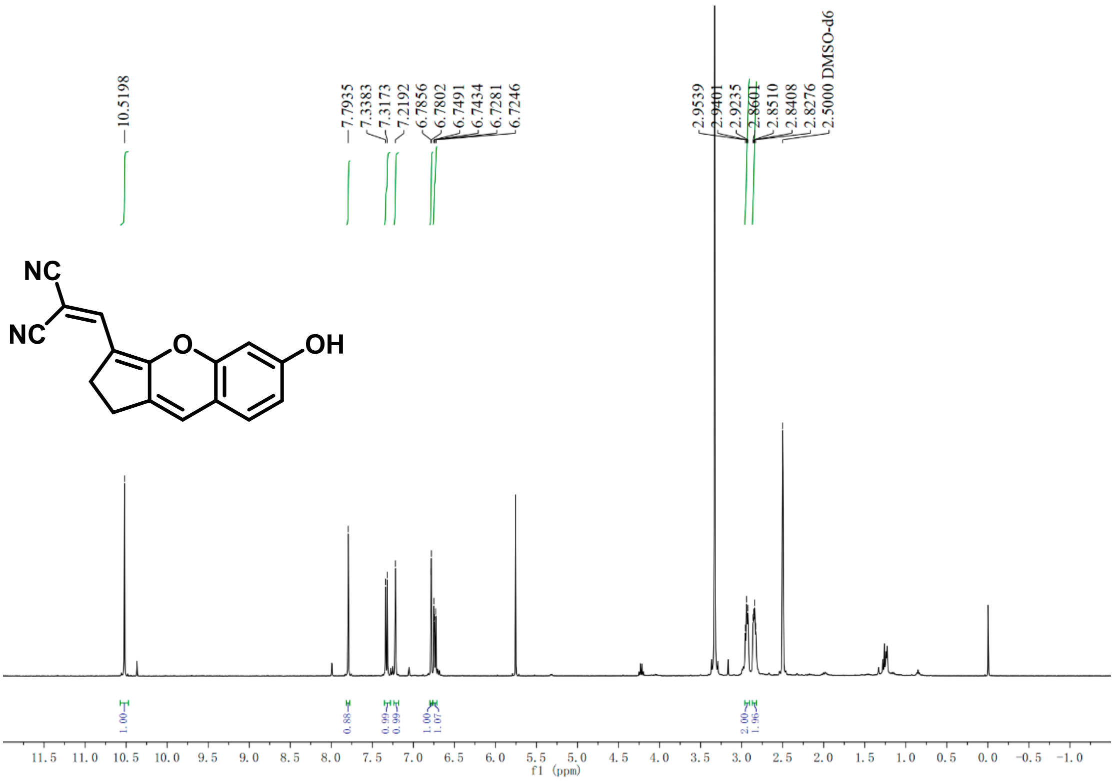

Figure S5. ^{1}H NMR spectrum (400 MHz, DMSO-d6) of **4**

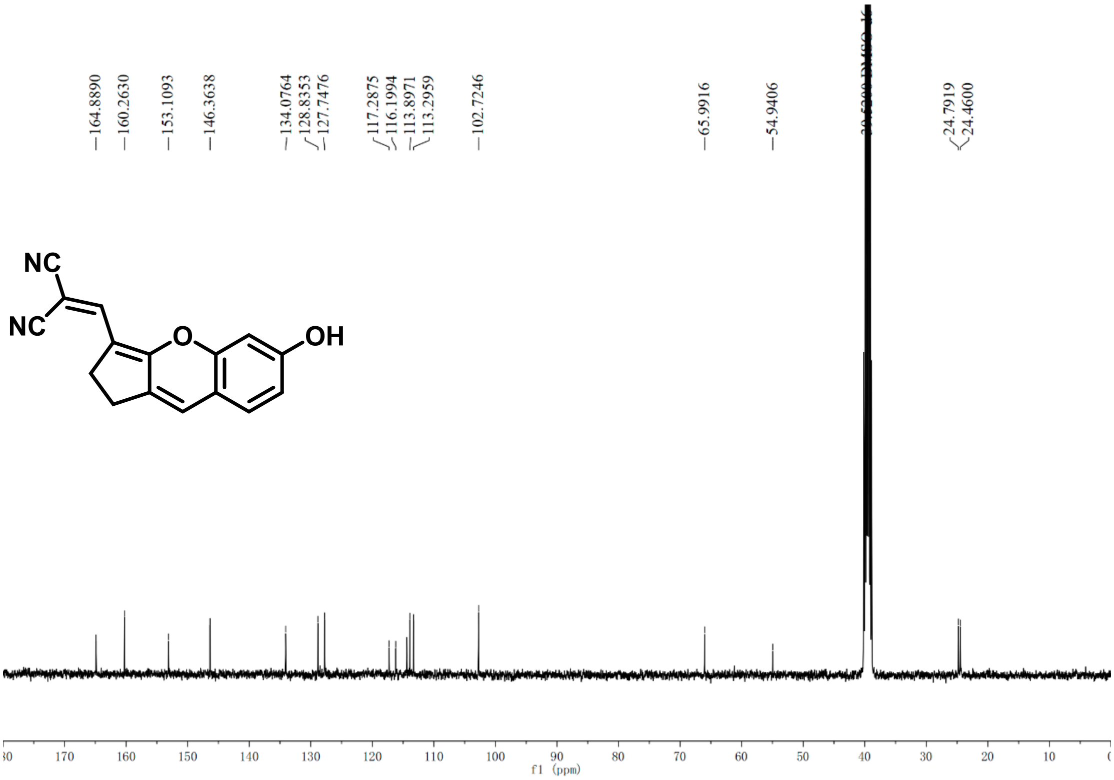

Figure S6. ^{13}C NMR spectrum (101 MHz, DMSO-d6) of **4**.

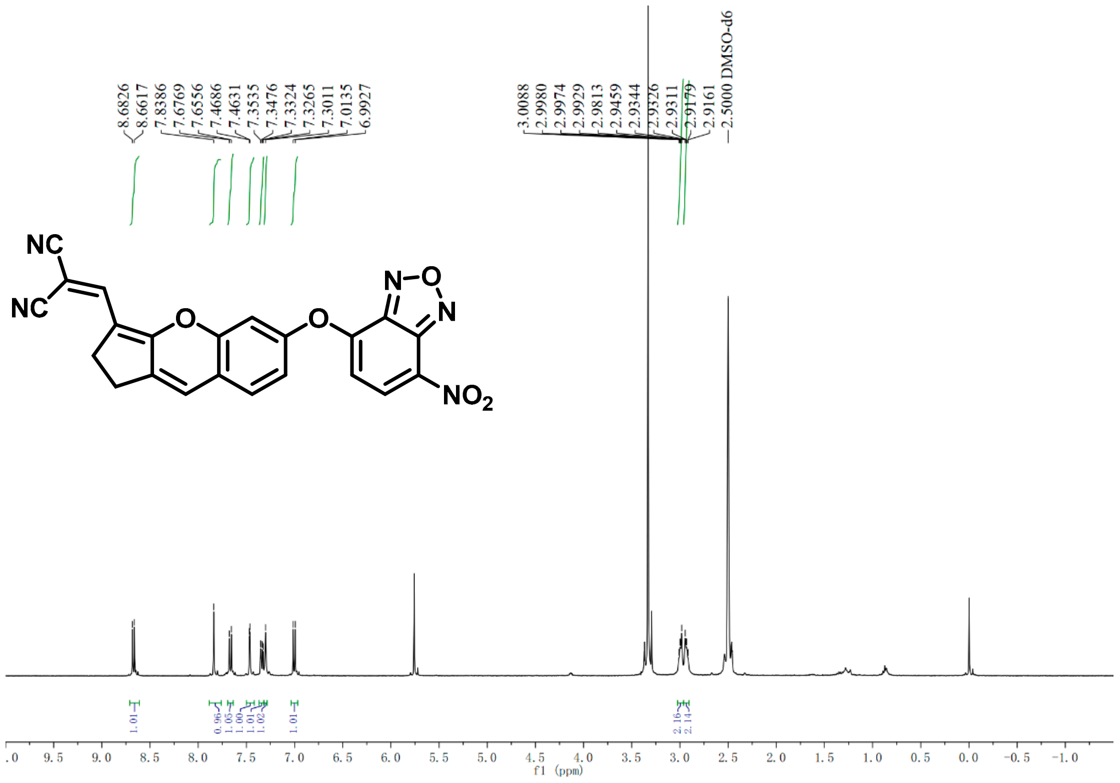

Figure S7. ^{1}H NMR spectrum (400 MHz, DMSO-d6) of **60T**

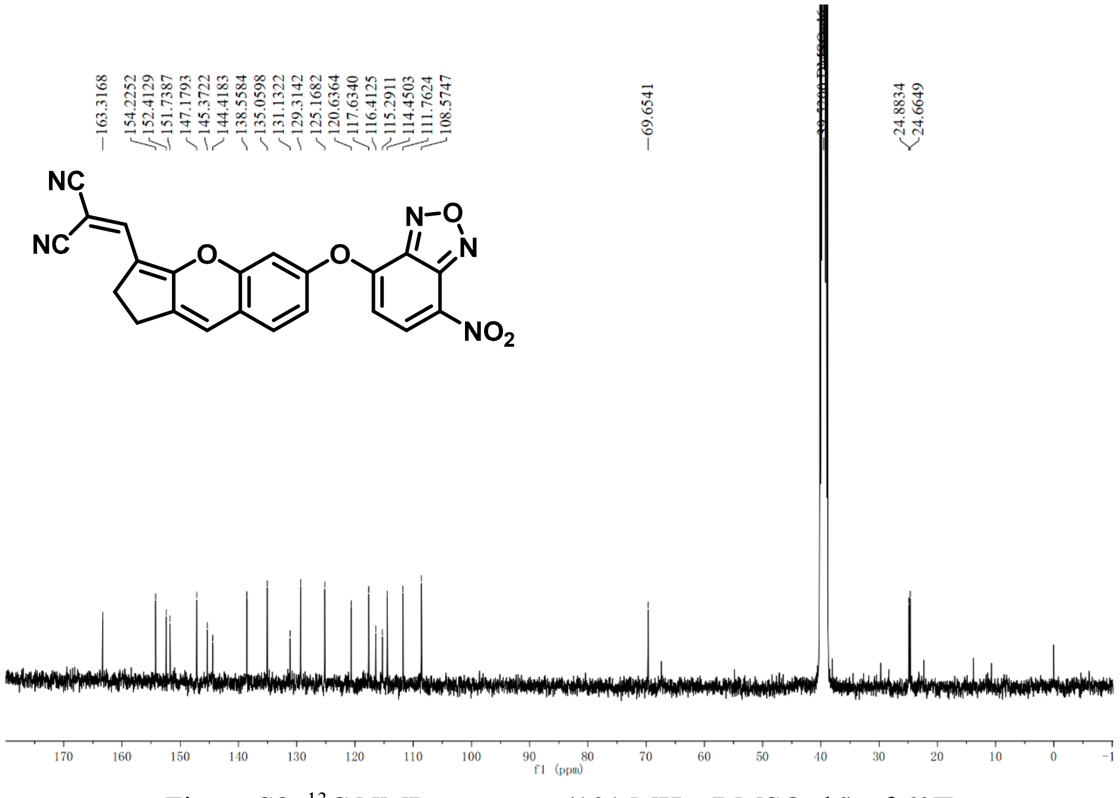

Figure S8. ^{13}C NMR spectrum (101 MHz, DMSO-d6) of **60T**
